# Development of Bio-Based and Recyclable Epoxy Adhesives by Modification with Thermoplastic Polymers

**DOI:** 10.3390/polym17020131

**Published:** 2025-01-08

**Authors:** Riccardo Miranda, Marco Luciano, Vincenzo Fiore, Antonino Valenza

**Affiliations:** Department of Engineering, University of Palermo, Viale delle Scienze, Edificio 6, 90128 Palermo, Italy; riccardo.miranda@unipa.it (R.M.); marco.luciano@unipa.it (M.L.); antonino.valenza@unipa.it (A.V.)

**Keywords:** epoxy adhesive, single-lap joint, recycling, disassembling, polyetherimide (PEI), poly-ε-caprolactone (PCL)

## Abstract

This paper deals with the design of novel epoxy adhesives by incorporating thermoplastic polymers such as polyetherimide (PEI) and poly(ε-caprolactone) (PCL) into a bio-based and recyclable epoxy resin, known as Polar Bear. The adhesives were characterized by their mechanical (quasi-static and dynamic) and rheological properties, thermal stability, and adhesion properties in single-lap joints tested at three different temperatures (i.e., −55 °C, 23 °C, 80 °C). The experimental results indicated that low PEI content substantially improved the mechanical performance and toughness of the adhesive, while preserving good processability. Nonetheless, exceeding 3% weight percentage adversely affected the adhesives’ mechanical resistance and workability. Conversely, while PCL addition enhanced the adhesives’ viscosity, it also decreased mechanical performance. However, its eco-friendliness offers potential for sustainable adhesive applications. It is worth noting that regardless of temperature, the modified adhesives consistently outperformed the commercial epoxy adhesive (DP-460), used as reference, in single-lap shear joint tests. Additionally, both PEI- and PCL-modified epoxy adhesives have demonstrated recyclability through a simple acid-based process, enabling joint disassembly and recycling of the adhesive into a thermoplastic polymer. Overall, the modified adhesives represent a promising eco-friendly, high-performance alternative for structural applications, aligning with sustainable and circular practices.

## 1. Introduction

Nowadays, the joining of structural elements has been the subject of study in various fields such as in the naval, aerospace, and automation industries. The use of adhesives, compared to other joining techniques like riveting or bolting, can be advantageous because it does not increase the weight of the component and does not require drilling, which could compromise the integrity of the structures, especially in large-sized ones. Thus, adhesives play a fundamental role in applications where lightweight structures and high mechanical performance are required or when it is necessary to join substrates of a different nature, such as metal and fiber-reinforced polymer composite [1,2,3]. Epoxy resins are widely used for bonding materials such as metals or composites in both the aerospace and naval sectors, as well as in the automotive industry [4,5,6,7,8]. Indeed, epoxy systems exhibit a high crosslink density, which positively influences mechanical performance. At the same time, a higher crosslink density improves resistance to chemical attack and increases the glass transition temperature (T_g_) but reduces the elongation at break, making the material brittle and thus limiting the range of applicability [9,10,11]. A way to improve the toughening of thermoset polymers, making the material less brittle, is adding thermoplastic polymers. Furthermore, the literature demonstrates that this addition could improve flexibility, impact resistance, and other mechanical properties without significantly compromising stiffness and adhesion [12,13,14,15]. For example, Chen et al. [16] investigated a novel approach to enhance the mechanical performance of epoxy resins by adding a thermoplastic material synthesized from diglycidyl ether of bisphenol A (DGEBA), namely, thermoplastic epoxy (TPE). In their study, they demonstrated that TPE/DGEBA blends could increase the tensile, flexural, impact, and fracture toughness properties, although they slightly negatively impacted thermal resistance by lowering the T_g_.

An example of a typically used thermoplastic polymer is polyetherimide (PEI), which offers an interesting opportunity to develop materials with superior characteristics [17,18]. PEI is widely used for its high mechanical and thermal properties, as well as its chemical stability in aggressive environments. Considering the possibility of using environmentally sustainable polymers, given current environmental issues, poly(ε-caprolactone) (PCL) could be an interesting alternative. PCL is a biodegradable thermoplastic that can provide greater flexibility to the material, as well as contributing to the sustainability of the final product [19,20]. Furthermore, PCL is considered a promising thermoplastic to integrate into epoxy resins due to its ability to promote physical crosslinking, as seen with hydrogen bonds, which optimize the system’s miscibility and enhance its workability [21]. Barone et al. [22] evaluated the thermomechanical properties of diglycidyl ether of bisphenol F (DGEBF) epoxy resin blended with PCL. The addition of PCL increases the toughness of the blend at the expense of the blend’s T_g_. To compensate for this reduction, DGEBF was mixed with a trifunctional epoxy resin, triglycidyl p-amino phenol (TGAP), which increased the T_g_ by increasing the crosslink density. This work demonstrated that through the addition of different PCL concentrations, the fracture resistance of the resulting epoxy/PCL blends was enhanced by the formation of a homogeneous miscible IPN (interpenetrated networks) with no phase separation. Moreover, the addition of thermoplastic polymers influences the viscosity of the epoxy system, increasing the range of applicability in the context of adhesives [23,24].

In such a context, Li et al. [25] studied the modification of epoxy resins with three different thermoplastics: methyl methacrylate block copolymer (BMG), polyphenylene oxide (PPO), and polysulfone (PSF). This work investigated those modifications on the curing process of the resin and its rheological properties, thermal stability, and mechanical performance. The results evidenced that BMG and PPO modifications increased processing capabilities due to lower viscosity, and the best toughening effect was observed with BMG, improving impact and flexural strength. Although PSF was thermally stable, it resulted in higher viscosity with less desirable mechanical properties.

Nowadays, due to environmental problems, the research focus must consider not only the mechanical performance and lightness but also the environmental impact of a material, with a focus on its sustainability and recyclability in the context of a circular economy.

In this regard, thermosetting polymer resins derived from renewable natural raw materials, and therefore defined as eco-sustainable or bio-based, could be a solution to the aforementioned requirements [26,27]. In recent decades, Connora Technologies, recently acquired by Aditya Birla Chemicals (Thailand), has developed a series of recyclable amine-based epoxy curing agents known as Recyclamines^®^. These molecules are characterized by a polyamine structure like that of traditional curing agents, but with a key innovation: the terminal amine groups are linked by a cleavable central group. This group is essential for recyclability as it allows for the conversion of the epoxy resin into a thermoplastic-based polymer through a chemical treatment performed in an acetic acid solution [28,29].

Dattilo et al. [30] explored the recyclability of a fully recyclable bio-based epoxy resin through a mild chemical recycling process. The thermomechanical properties of the recycled product were analyzed using DSC and DMA, while thermogravimetric analysis confirmed the stability of the recycled polymer. This study highlighted the potential to make the recycled product available for new applications, enhancing its reuse.

This work aims to design and characterize eco-sustainable structural adhesives by modifying a partially bio-based and recyclable epoxy system with the addition of thermoplastics polymers. These innovative systems were compared to a commercial structural adhesive to evaluate their applicability. In addition to the preliminary characterization of the proposed adhesives, their feasibility in adhesive joints was evaluated by assessing the single-lap shear strength of the joints at various temperatures. Finally, the chemical recyclability of the modified epoxy systems was evaluated.

## 2. Materials and Methods

### 2.1. Materials

This study aims to modify the Polar Bear epoxy system (purchased from R*Concept, Barcelona, Spain) by adding thermoplastic polymers to evaluate its potential as a recyclable structural adhesive. The neat epoxy system can be considered partially bio-based since, according to its datasheet, its biocarbon content is more than 28%. It is obtained by mixing the epoxy monomer (part A) with the hardener Recyclamine™ R*101 (part B) in a 100:22 weight ratio. Recyclamine™ R*101 is a recyclable amine hardener characterized by the presence of acid-cleavable amine groups in its chemical structure, enabling chemical recyclability. The Polar Bear epoxy system was modified by evaluating the effect of adding two thermoplastic polymers: polyetherimide (PEI–Mn 20000) supplied by Goodfellow Cambridge Ltd. (Huntingdon, UK) and poly-ε-caprolactone (PCL–Mn 80000) supplied by Sigma-Aldritch (St. Louis, MO, USA).

A commercial two-component epoxy adhesive (Scotch-Weld™ DP460 supplied by 3M™, Saint Paul, MN, USA) served as a comparison reference for the preliminary rheological, mechanical, and dynamic–mechanical testing of the proposed adhesive systems.

The metal substrate to which the adhesive was applied was an aluminium alloy 6061 with a nominal thickness of 3 mm (Table 1).

### 2.2. Preparation of Adhesives

The Polar Bear epoxy resin was modified by evaluating the impact of incorporating two thermoplastic polymers: poly-ε-caprolactone (PCL) and polyetherimide (PEI). PCL is a biodegradable thermoplastic with a simple linear structure, a low melting point, and a glass transition temperature below room temperature, while PEI is a high-performance engineering polymer known for its excellent thermal stability, mechanical strength, and good chemical resistance.

PCL pellets were directly mixed with the epoxy monomer at weight percentages of 5%, 10%, 15%, and 20% because of their high solubility in epoxy systems. The mixture was heated to 90 °C for 4 h while being stirred with a magnetic stirrer to obtain a homogeneous solution [31]. Finally, after cooling to 20 °C, the hardener was added to the mix, and the curing process took place under the same conditions as in the previous case.

In contrast, PEI was added to the neat epoxy system at concentrations of 1%, 2%, 3%, 4%, and 5% by weight of the monomer (part A). Increasing concentrations of PEI significantly increases the viscosity of the resulting system. To avoid this, the concentration was limited to 5% by weight.

In more detail, this engineering thermoplastic polymer was first dissolved in dichloromethane and then added to the epoxy monomer. The solution was heated at 90 °C for 3 h to completely evaporate the dichloromethane and ensure uniform mixing of the monomer with the PEI [17,18]. Afterwards, the system was cooled to 20 °C before the hardener was added. The curing process was carried out at room temperature for 24 h, followed by a post-cure of 3 h at 100 °C.

Table 2 lists the prepared samples, the adopted nomenclature, and the relative concentration of each constituent per 100 parts of epoxy monomer.

### 2.3. FT-IR Analysis

Fourier transform infrared spectroscopy (FT-IR) analysis was carried out using a Perkin Elmer (Waltham, MA, USA) spectrometer model Spectrum II to assess the presence of the thermoplastic polymers after mixing with the epoxy system. The absorbance spectrum was recorded in the frequency range of 450–4000 cm^−1^, with a resolution of 4 cm^−1^.

### 2.4. Rheological Characterization

Rheological tests were conducted to evaluate the viscosity of the modified epoxy systems and compare them to the viscosity value of the commercial epoxy adhesive DP-460. In this context, an ARES-G2 rotational rheometer from TA Instruments (New Castle, DE, USA) was used in shear flow. All tests were carried out in parallel plates with a gap of about 1.5 mm and a diameter of 25 mm at room temperature, setting the rotational speed at 30 rad/s. All viscosity measurements were taken 5 min after the hardener was mixed into the epoxy monomer–thermoplastic blend to assess the adhesive’s initial workability.

### 2.5. Mechanical Tests

Three-point bending characterization was used to evaluate the mechanical properties of the compared materials, such as flexural strength and modulus. Five samples (60 mm long, 13 mm wide, and 3 mm thick) of each adhesive type were tested using a Zwick–Roell universal testing machine model Z005 (Ulm, Germany) equipped with a 5 kN load cell. All tests were conducted in accordance with ASTM D790 standards [32], using a crosshead speed equal to 1.5 mm/min and a support span of 50 mm, respectively.

Dynamic mechanical thermal analysis (DMTA) was conducted in tensile mode in accordance with the ASTM D4065 standard [33] using a dynamic mechanical analyzer DMA +150 by Metravib (Limonest, France). Three prismatic samples (4 mm × 46 mm) per condition were tested from 25 °C up to 150 °C, with a heating rate of 10 °C/min. The dynamic displacement and frequency were set at 1 × 10^−5^ m and 1 Hz, respectively.

### 2.6. Morphological Analysis

To assess the homogeneity of the system, a FEI (Hillsboro, OR, USA) Quanta 200 FEG scanning electron microscope (SEM) was used to examine the fracture surfaces of polymeric samples after three-point bending.

Aluminium stubs were used, with thin carbon tape applied to the samples. The fracture surfaces were sputter-coated with gold prior to fractographic examination. The images were obtained with an accelerating voltage of 10 kV.

### 2.7. Joints Manufacturing and Testing

The single-lap shear test was selected to comparatively evaluate the performance of epoxy systems as adhesive for joints between two aluminum alloy 6061 substrates. Indeed, this test is useful for verifying the adhesion between two aluminum substrates and for estimating the mechanical properties, specifically average shear strength, of the cured adhesive. To manufacture the adhesive joints, the metallic surfaces were previously sandblasted with 80-grit sandpaper. An amount of approximately 0.4 g of epoxy adhesive was applied between the two surfaces, ensuring an average adhesive layer thickness of 0.15 mm. The overlap area between the two substrates measured 25 mm × 25 mm. Subsequently, the samples were subjected to the same curing and post-curing cycle, described in Section 2.2.

Lap-shear tests were performed on five samples of each investigated adhesive using a universal testing machine model ETM-C by Wance (Shenzhen, China), equipped with a 50 kN load cell and a climatic chamber. The crosshead speed was set as 1.6 mm/min following the ASTM D1002 standard [34]. The mechanical tests were conducted at −55 °C, 25 °C, and 80 °C to evaluate the adhesion strength of the investigated epoxy systems under varying environmental conditions. To ensure temperature uniformity, each sample was kept at the chosen temperature for 15 min to allow the system to reach thermal equilibrium before performing the mechanical test.

### 2.8. Disassembling of Joints and Recycling Process

The disassembling process was carried out by dipping the adhesive joints in a solution of 75% *v*/*v* of acetic acid at 90–100 °C for 3 h. Once the adhesive was dissolved and the metallic substrates were completely separated, they were washed in deionized water. The recycling process starts with the same procedure as the dissolving process. The process was conducted in detail using 10 g of each type of cured adhesive.

**Figure 1:**
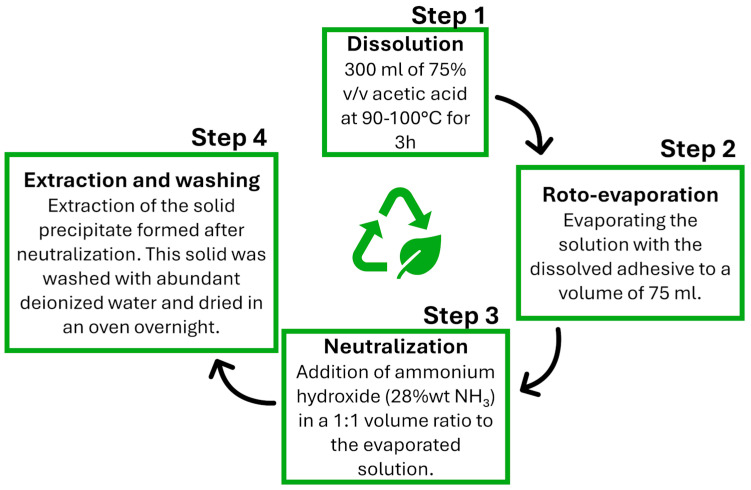
Schematic representation of the recycling process.

The recycling process is inspired by the procedure of Saitta et al. [35] and assesses the feasibility of generating recycled products, even when incorporating thermoplastic polymers such as PEI and PCL into Polar epoxy resin. As already mentioned, the process begins with the dissolution of 10 g of epoxy-based adhesive in 300 mL of a stirred 75% *v*/*v* acetic acid solution at 90–100 °C for 3 h until complete dissolution is achieved. This step is crucial as it facilitates the cleavage of the cross-linked network of the epoxy resin. Acetic acid acts as a solvent that helps in breaking the covalent bonds within the epoxy matrix. The use of a special amine with a cleavable ketal group enhances this process, allowing for selective cleavage under mild acidic conditions. Afterwards, the excess of acetic acid evaporated until the solution reached a volume of 75 mL, obtaining a concentrated solution. The concentrated solution is then neutralized using an ammonium hydroxide solution. This neutralization step is essential for precipitating the cleaved epoxy resin from the solution. The ammonium hydroxide is prepared by mixing water and ammonium hydroxide (28–30% NH_3_ by weight) in 1:1 ratio. The recycling process is designed to minimize energy consumption, particularly during the drying phase and the use of solvents. Upon stirring, a solid precipitate formed and gradually agglomerated into a reusable thermoplastic polymer, demonstrating the success of the recycling process. The recycling mechanism described (see schematic representation in Figure 1) combines both chemical and physical processes to effectively break down and recover materials from bio-based epoxy thermosets.

## 3. Results and Discussion

### 3.1. Epoxy-Based Adhesives

#### 3.1.1. FT-IR 

**Figure 2:**
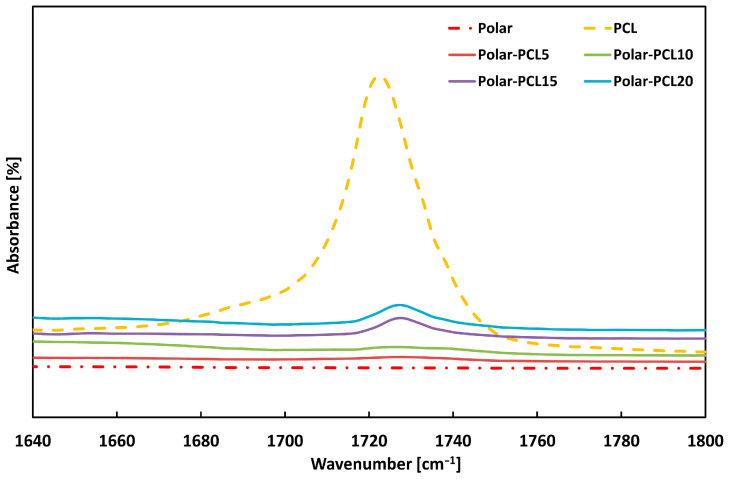
FTIR spectra of neat Polar, neat PCL, and Polar-PCL blends with varying PCL content.

**Figure 3:**
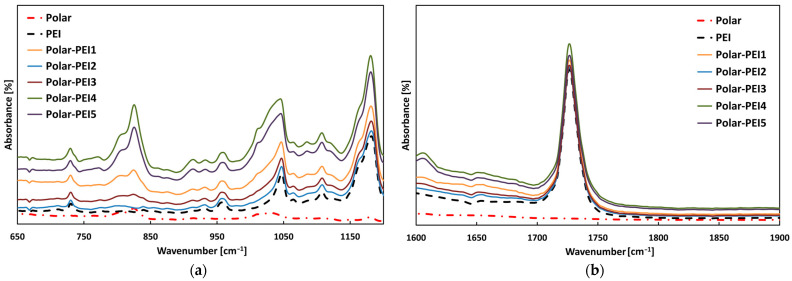
FTIR of neat Polar, neat PEI, and Polar-PEI blends with varying PEI content spectra in the regions of (**a**) 650–1200 cm^−1^ and (**b**) 1600–1900 cm^−1^.

The FT-IR spectra of the neat polymers and related blends are shown in Figure 2 and Figure 3.

It is worth noting that FT-IR results provide evidence for the presence of both PCL and PEI within the Polar epoxy system. More specifically, Figure 2 clearly shows the shift of the peak at 1723 cm^−1^ for neat PCL—due to characteristic elongation of carbonyl groups (-C=O)—to the higher frequency of 1735 cm^−1^ for Polar-PCL with a varying PCL percentage. This indicates the formation of the interchain hydrogen bonding involving the carbonyl group of PCL and the hydroxyl groups of epoxy [36,37]. On the other hand, two peaks centred at 725 cm^−1^ and 1100 cm^−1^, associated with the deformation of the imide ring, are visible in the spectra of the Polar-PEI blends shown in Figure 3a [38]. Another proof of the presence of PEI in the Polar epoxy system is shown in Figure 3b, where there is the peak at 1720 cm^−1^ associated with the symmetrical stretching vibrations of the imide groups [38].

#### 3.1.2. Rheology

Rheological tests were conducted to identify the most suitable epoxy resin–thermoplastic mixture for the bonding application. As detailed in the experimental section, the initial viscosity of each system (measured immediately after hardener addition to the epoxy monomer–thermoplastic blend) was recorded as an indicator of the adhesive’s initial workability.

**Figure 4:**
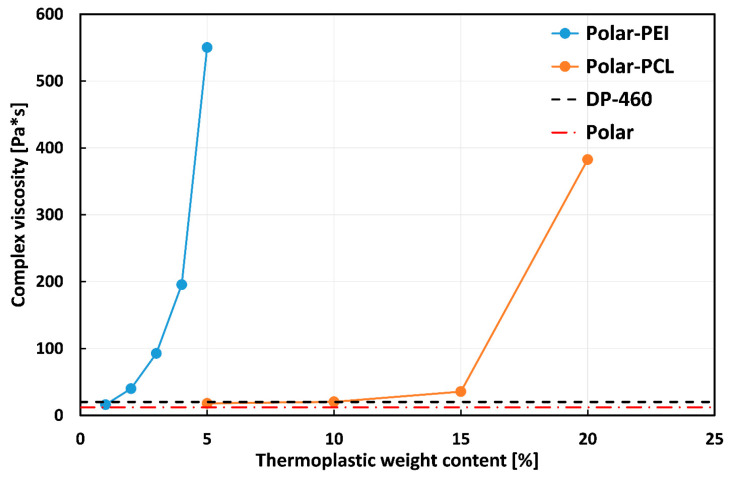
Complex viscosity values measured at the onset of the curing process for each polymeric system, varying thermoplastic content.

The results, shown in Figure 4, compare the rheological behavior of the PEI- and PCL-modified epoxy systems with that of a commercial adhesive (DP-460), chosen as reference.

As expected, the workability of the epoxy system decreased with the increasing thermoplastic content. In more detail, by adding small amounts of PEI to the epoxy system, the initial viscosity value increases exponentially from 12 Pa∙s (Polar) to 550 Pa∙s (Polar-PEI5). It is quite evident, by observing this graph, that 5% by weight of PEI added to the polar epoxy resin appears to be the upper limit. Beyond this point, the material exhibits very high viscosity (i.e., it acquires a rubbery consistency), which makes it unsuitable for bonding applications. Considering 1% and 2% PEI, the viscosity of the modified systems approached that of the commercial epoxy-based adhesive DP-460 (i.e., 20 Pa∙s). This favored spreadability while maintaining a consistency similar to that of an epoxy adhesive. On the other hand, the addition of PCL only generates an exponential trend after a considerable amount of added polymer. In fact, up to 15% of PEI, the viscosity of modified Polar systems remains very close to that of commercial adhesive, making them suitable for applications such as adhesives. Upon reaching 20% PCL, the adhesive system acquires a rubbery consistency, as with the addition of 5% PEI. In such a context, it is possible to observe that epoxy systems modified with the same weight of thermoplastic polymers (i.e., Polar-PEI5 and Polar-PCL5) exhibit opposite rheological behavior. More specifically, the viscosity of the Polar-PEI5 system (550 Pa·s) is approximately 30 times higher than that of the Polar-PCL5 system (i.e., 18 Pa·s), which is probably due to a stronger chemical interaction between the epoxy network and PEI compared to PCL.

These results demonstrate that the addition of a small amount of thermoplastic polymer to this innovative epoxy system (Polar Bear) significantly increases viscosity, thereby altering its processability. Larger quantities resulted in the formation of macroscopic defects, primarily due to difficulties in mould filling, thereby compromising the quality of the resulting materials. Without the addition of thermoplastics, the viscosity of neat epoxy resin is too low (i.e., 12 Pa∙s) for use as an adhesive in secondary bonding joints. However, by adding 1–2% PEI or 5–10% PCL, the viscosity can be increased to a level suitable for adhesive applications.

#### 3.1.3. Three-Point Bending

The mechanical properties of the compared polymeric systems evaluated through three-point bending tests are listed in Table 3.

The results indicate that the addition of 1% and 2% by weight of PEI to the neat epoxy resin (Polar) do not affect its flexural strength, while yielding a 62.4% and 68% increase, respectively, compared with the commercial epoxy-based adhesive DP-460. Compared with neat epoxy resin, the Polar-PEI1 and Polar-PEI2 systems exhibit an 8.7% and 37% increase, respectively, in deformation at break. This finding highlights the high miscibility of a low content of polyetherimide in the epoxy resin and its cleavable amine, thus leading to the enhanced ductility of the resulting systems without compromising their mechanical strength, as supported by the surface morphology shown in Figure 8. Beyond a 2% PEI addition, the Polar-PEI systems’ mechanical strength declines due to the excess PEI, which induces macroscopic defects and brittle behavior, leading to reduced deformation at break. In particular, the addition of 5% PEI by weight reduced the flexural strength of the resulting system (Polar-PEI5) by half compared to the neat epoxy resin (−18.7% in comparison to that of the commercial adhesive DP-460).

Conversely, the incorporation of PCL results in a decrease in flexural strength, ranging from 30% to 40% lower than DP-460 and from 50% to 64% lower than the neat epoxy resin. These decrements in the mechanical strength of the adhesive can be mainly ascribed to the quite-low mechanical properties of the PCL, such as low modulus and tensile strength [39]. Overall, Polar-PCL systems exhibited increased brittleness compared to neat epoxy resin, as evidenced by the decreased deformation at break.

Regarding the stiffness of the compared systems, adding thermoplastic polymers (PEI or PCL) to the epoxy network consistently decreased the flexural modulus of the tested samples compared to the neat epoxy, regardless of the polymer type or its weight content. On the other hand, all the modified epoxy systems (Polar-PEI and Polar-PCL) showed a higher flexural modulus than the commercial epoxy-based adhesive DP-460 (except for Polar-PCL20 system).

The effect of adding thermoplastic polymers such as PEI and PCL on the mechanical behavior of the proposed epoxy-based systems was also evaluated by calculating their toughness, which is proportional to the area under the flexural stress–strain curves, as suggested by the literature [40,41]. Indeed, toughness reflects a material’s ability to absorb energy before failure. As a result, tougher materials typically exhibit higher strength and/or larger strain at break than softer ones.

In this paper, toughness values for all investigated polymer systems were calculated using the method described by Calabrese et al. [42].

**Figure 5:**
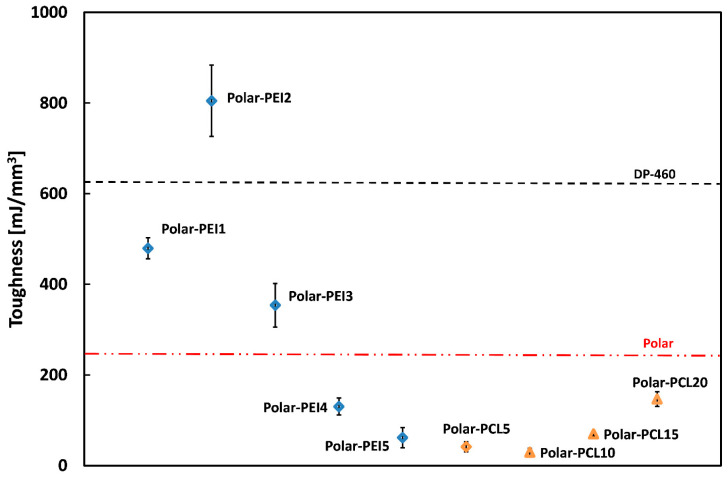
Toughness values of the proposed epoxy adhesives compared to commercial adhesive DP-460.

As evidenced in Figure 5, the commercial adhesive DP-460 shows high strain at break and limited flexural strength values, resulting in high toughness due to its high deformability. The low toughness values exhibited by Polar-PCL blends (i.e., lower than the neat epoxy) confirm that the presence of PCL in the epoxy network renders these systems more brittle (i.e., characterized by a high modulus but low strength and strain at break values). Conversely, the Polar-PEI systems display rubber-like behavior for compositions up to 3% PEI by weight.

Notably, the Polar-PEI2 blends show very high toughness values (i.e., +29% compared to DP-460 and +220% compared to neat epoxy), making them well-suited for adhesive bonding applications. Beyond this content threshold, the resulting systems evidence reduced toughness, confirming that excessive PEI induces defects in the epoxy matrix, leading to brittle materials. These results are consistent with the SEM images shown in Section 3.1.5.

**Figure 6:**
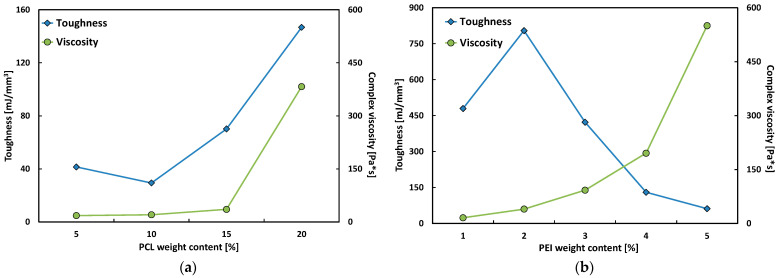
Toughness versus viscosity trends for (**a**) Polar-PCL and (**b**) Polar-PEI systems.

Additional considerations may arise from a comparative analysis of toughness and complex viscosity trends for all investigated polymeric systems, as depicted in Figure 6.

First, it is worth noting that the viscosity of both systems increases with increasing thermoplastic content, whereas the toughness trend is a function of the specific thermoplastic used. By considering Polar-PEI samples, the toughness increases for PEI content up to 2%, resulting in a tough, epoxy-modified system. However, further increasing the thermoplastic content led to a more brittle behavior of the resulting blends. Therefore, epoxy systems modified with the addition of up to 2% by weight of PEI emerge as the optimal choice for adhesive bonding applications. They combine ideal viscosity, ensuring high workability, as demonstrated by comparison with the commercial adhesive DP-460. Additionally, their high flexural strength and deformability contribute to excellent toughness.

On the other hand, the Polar-PCL system shows a growing trend for both toughness and viscosity as the weight content of the added thermoplastic increases. This leads to an undesirable outcome, as the goal of achieving good mechanical performance can be reached by simply adding high PCL content to the epoxy resin. However, this approach results in excessive viscosity, which hinders the workability of the adhesive for bonding applications. Therefore, the best compromise in this case lies in the middle, accepting a few losses in mechanical performance while maintaining acceptable workability.

Furthermore, it is worth noting that at the overlapping concentration range (i.e., 5%), the compared epoxy systems (i.e., Polar-PEI5 and Polar-PCL5) exhibit very different viscosities but similar mechanical performance. As stated in the previous section, it can be assumed that, from a rheological point of view, the chemical interaction between the added thermoplastic polymer (i.e., PEI or PCL) and the Polar Bear epoxy system plays a key role in the behavior of the modified systems.

#### 3.1.4. Dynamic Mechanical Analysis

**Figure 7:**
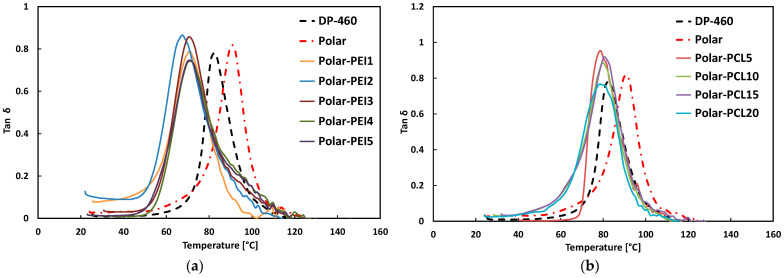
Damping factor (tanδ) trends of (**a**) Polar-PEI and (**b**) Polar-PCL systems compared to neat epoxy and commercial adhesive DP-460.

Figure 7 shows the effect of PEI and PCL addition on the damping factor (tanδ) trend of the resulting modified epoxy systems at varying temperatures.

The analysis of the dynamic behavior (Figure 7a) reveals that the curves for the Polar-PEI samples are shifted to lower temperatures compared to those of the commercial adhesive DP-460 and neat epoxy resin. This means that all Polar-PEI samples exhibit lower T_g_ values compared to the commercial adhesive, as reported in Table 4. The lower T_g_ values suggest that the introduction of Polar-PEI results in reduced thermal resistance compared to DP-460, making the samples more sensitive to temperature variations.

All Polar-PCL samples exhibit a slight shift of the tan δ curve to lower temperatures (Figure 7b). This behavior suggests that the addition of PCL does not significantly affect the T_g_ in the same manner as PEI. In other words, PCL does not seem to influence the glass transition temperature as much, indicating less interaction with the neat epoxy resin compared to PEI. In general, the addition of both PCL and PEI reduces the T_g_ values of the modified epoxy system. This plasticization effect, attributed to the presence of thermoplastic polymers within the thermoset network, is consistent with previous studies [11,16,43,44].

#### 3.1.5. Morphology

**Figure 8:**
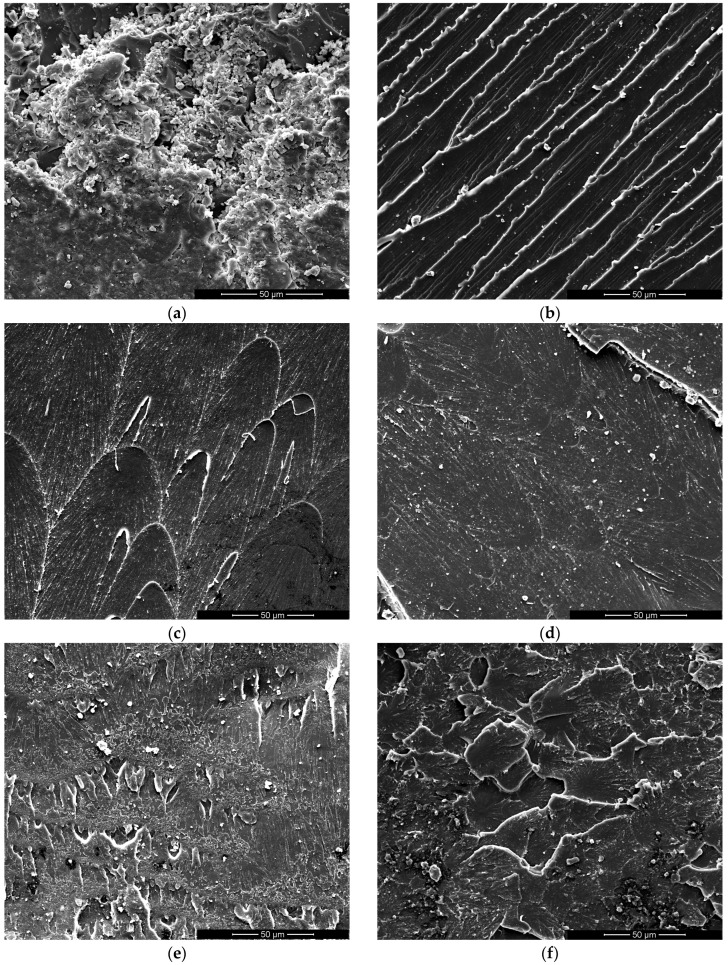
Fractured surfaces of Polar-PEI samples with varying thermoplastic content: (**a**) Polar; (**b**) Polar-PEI1; (**c**) Polar-PEI2; (**d**) Polar-PEI3; (**e**) Polar-PEI4; (**f**) Polar-PEI5 systems.

**Figure 9:**
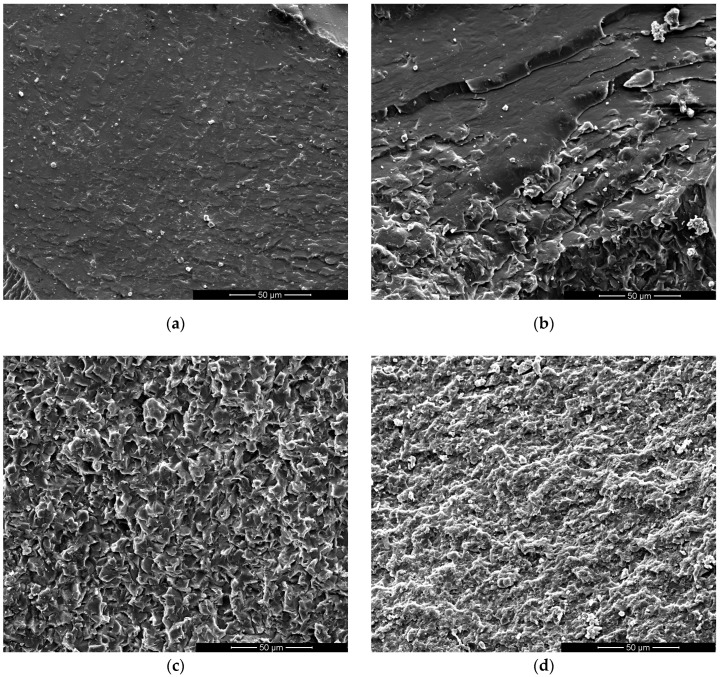
Fractured surfaces of Polar-PCL samples with varying thermoplastic content: (**a**) Polar-PCL5; (**b**) Polar-PCL10; (**c**) Polar-PCL15; (**d**) Polar-PCL20 systems.

SEM images of the fractured bending samples reveal a uniform distribution of both thermoplastic polymers (PCL and PEI) within the epoxy network. Indeed, no phase separation between the thermoset matrix and the thermoplastic phases (PEI or PCL) is evident in Figure 8 and Figure 9. Moreover, morphological analysis clearly demonstrates the impact of the addition of thermoplastic polymers on the epoxy system’s fracture mechanisms. Figure 8a shows that the fracture surface of the neat epoxy resin (i.e., Polar) is quite rough, indicating an intermediate value of deformations before rupture that justified the toughness values in the previous paragraph. In the literature, a rougher fracture surface is often associated with enhanced toughness [15,18,37].

The incorporation of polyetherimide (PEI) into the epoxy network yields a somewhat different behavior, as evidenced in Figure 8. The fractures of Polar-PEI samples did not evidence a rough surface, as visible in neat epoxy resin, but exhibited uniform linear markers. In the literature, this behavior is associated with a significant plastic deformation occurring within the material before final fracture [15]. These findings corroborate the higher toughness values exhibited by Polar-PEI1, Polar-PEI2, and Polar-PEI3 compared to Polar. Furthermore, this behavior in Polar-PEI2 even leads to an increase in toughness compared to DP-460. However, the fracture surfaces of Polar-PEI4 and Polar-PEI5 (Figure 8e,f) closely resemble that of neat epoxy resin (Figure 8a), justifying the toughness values reported in Figure 6.

Conversely, for Polar-PCL5 and Polar-PCL10, as shown in Figure 9a,b, the surface appears smoother, which corresponds to lower toughness.

As the percentage of PCL increases, the surface roughness also increases (Figure 9c,d), accompanied by an improvement in toughness. However, the toughness of Polar-PCL remains lower than that of neat epoxy resin (i.e., Polar) and the commercial adhesive DP-460.

### 3.2. Single-Lap Joint Tests

After the preliminary evaluation of the main properties of the modified epoxy systems, samples demonstrating the best correlation between workability and mechanical performance were selected as adhesives for single-lap joints between two aluminium alloy 6061 substrates. Among Polar-PEI systems, only those with 1% and 2% PEI additions outperformed all characteristics due to their viscosity, comparable to the commercial adhesive DP-460, and they also exhibited high mechanical performance and a good viscoelastic response.

On the other hand, despite their low mechanical response, among the PCL-modified epoxy resins, only Polar-PCL5 and Polar-PCL10 were selected as adhesives for single-lap joints due to the very low workability (i.e., high viscosity) exhibited by the epoxy systems modified with 15% and 20% by weight of polycaprolactone. The selected epoxy systems were compared to the commercial DP-460 as an adhesive for single-lap joints, which were tested under three different temperatures (i.e., −55 °C, 23 °C. and 80 °C) to gain a better understanding of the proposed adhesive at varying conditions.

**Figure 10:**
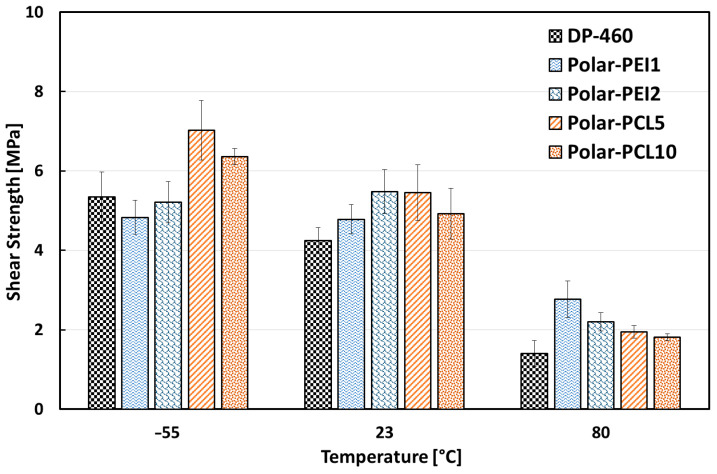
Single-lap joints’ shear strength at varying temperature conditions.

Figure 10 shows the average shear strength and corresponding standard deviations of single-lap joints at 23 °C, −55 °C, and 80 °C.

All joints fabricated using modified adhesives exhibit superior performance compared to those employing commercial adhesive DP-460 at room temperature (23 °C). In this temperature condition, Polar-PEI2 and Polar-PCL5 joints exhibit the highest increases (+22.4% and +22.1%, respectively), followed by Polar-PCL10 (+13.6%) and Polar-PEI1 (+11.2%), in comparison to DP-460-based joints. At higher temperatures (i.e., 80 °C), all specimens show decreases in shear strength. This is likely due to the proximity of this temperature to the glass transition temperature values (T_g_) of the polymeric adhesive systems. DP-460-based joints experienced the most significant decrease in shear strength (−67%)—whereas Polar-PEI1 showed the least (−42.2%), followed by Polar-PEI2 (−60%), Polar-PCL10 (−63.3%), and Polar-PCL5 (−64.4%)—when compared to their respective values at room temperature. Similar to their performance at room-temperature, the modified adhesives outperformed commercial DP-460, even at 80 °C.

By considering the mechanical behavior of joints at sub-zero conditions (−55 °C), all compared adhesives demonstrated quite-high mechanical resistance, indicating their suitability for extreme conditions such as those encountered in high-altitude flights. Under these environmental conditions, the mechanical response of joints bonded with Polar-PEI epoxy adhesives is comparable to that of joints bonded with the commercial adhesive DP-460. However, surprisingly, Polar-PCL-based joints exhibit the highest mechanical resistance values of all the joints investigated. This behavior can be attributed to the temperature dependence of the maximum resistance of the polymer used as an adhesive of the bonded joint and the fracture energy. This parameter depends on both the Van der Waals and covalent bonds within the polymer structure. The stiffness of the Van der Waals bonds decreases with increasing temperature and becomes negligible near the glass transition temperature (T_g_). This could be the reason for the monotonic decrease in maximum adhesive resistance with increasing temperature, which is directly correlated with the joint’s shear strength [45].

In conclusion, regardless of temperature conditions, Polar-PEI and Polar-PCL modified systems can be considered as suitable alternatives to the commercial adhesive DP-460. Indeed, these systems exhibit very high performance as adhesives for single-lap joints, supporting their applicability as a structural adhesive in different environmental conditions.

### 3.3. Disassembly and Recycling

**Figure 11:**
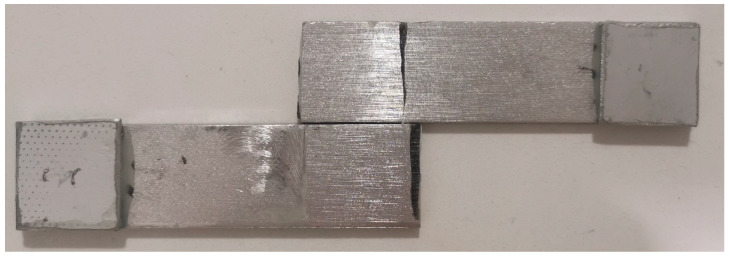
Metallic substrates separated after joint disassembly.

The utilization of recyclable epoxy systems as adhesives allows for the disassembly of joints and the recycling of polymeric materials. To achieve this, joints were immersed in an acid solution following the procedure outlined in Section 2.8. As shown in Figure 11, this process resulted in the complete separation of the metallic substrates, with no trace of adhesive on their surface.

**Figure 12:**
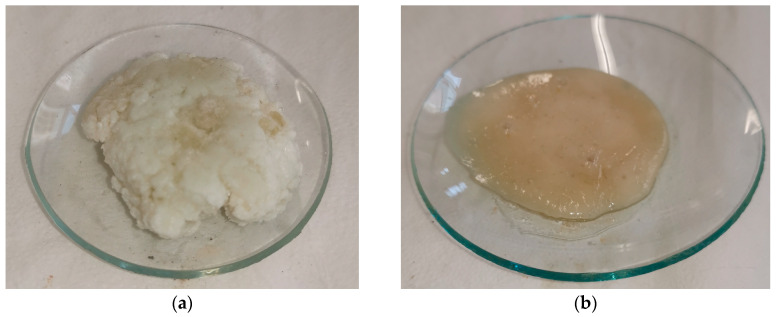

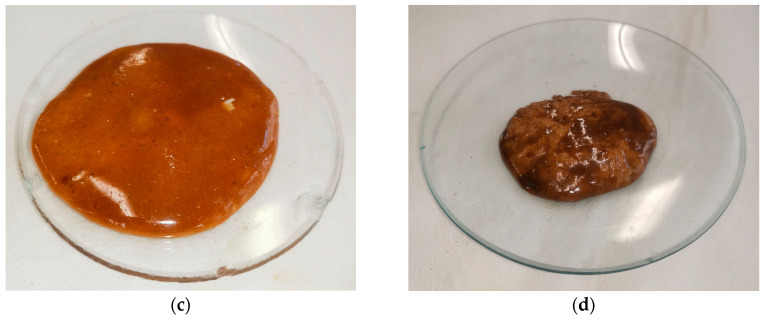
Thermoplastic polymers extracted after the recycling process of (**a**) Polar-PCL5; (**b**) Polar-PCL10; (**c**) Polar-PEI1; (**d**) Polar-PEI2 epoxy systems.

Furthermore, thermoplastic recycled polymers were obtained as a precipitate, as shown in Figure 12. This was achieved through the amine of the Polar system, which contained a cleavable ketal group within its structure. This design enables the selective cleavage of the cured epoxy resin network under mild acidic conditions. When the cross-linked resin is treated with an acid, such as acetic acid, the cleavable ketal group facilitates the breaking of the ethereal bridges in the polymer network. This leads to the depolymerization of the thermoset material, transforming it into a recycled polymer with thermoplastic properties.

These results confirm the feasibility of modified epoxy systems as recyclable structural adhesives.

**Figure 13:**
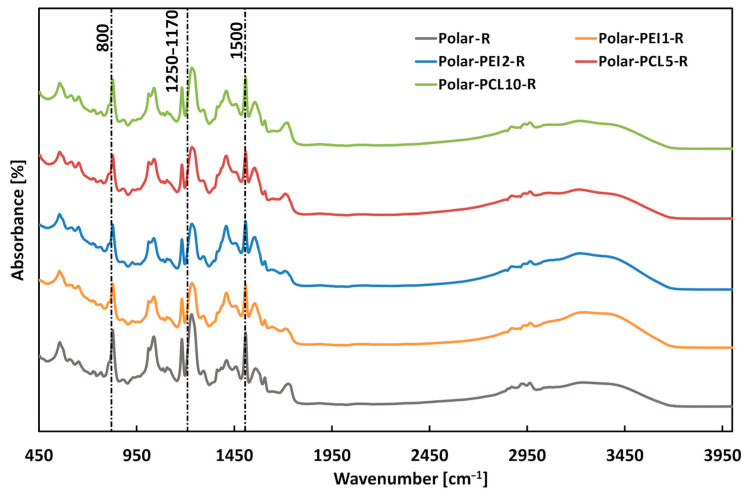
FTIR spectra of recycled thermoplastic polymers.

Finally, the FTIR spectra of the recycled polymers were analyzed. As visible in Figure 13, the FTIR spectra are nearly identical, indicating that the addition of PCL or PEI to the epoxy resin does not significantly modify the chemical composition of the thermoplastic polymer obtained after the recycling process. As reported in the literature [30], the resulting thermoplastic polymer contains functional groups typical of the neat resin, i.e., the vibrations of aryl–alkyl–ether groups (1250 to 1170 cm^−1^) and those of aromatic rings (1500 cm^−1^) and 1,4-disubstituted aromatic rings (800 cm^−1^). This result depends on the cleavage mechanism that took place only at the cross-linked point, keeping the chemical structure outside the cross-link unaffected.

## 4. Conclusions

Adhesive joining is a critical process in industrial applications, but conventional materials, such as epoxy resins, raise environmental concerns due to their non-recyclable nature, which makes adhesive joints non-disassembleable. In this context, this paper presents more eco-friendly solutions based on the use of a partially bio-based and recyclable epoxy resin (named Polar Bear). This resin was modified by adding thermoplastic polymers—polyetherimide (PEI) and poly(ε-caprolactone) (PCL)—to achieve a viscosity suitable for industrial applications while maintaining adequate mechanical performance.

The main findings of this study can be summarized as follows:

- Polar-PEI adhesives with low thermoplastic content (1–2%) exhibited increased viscosity without compromising mechanical strength, compared to the commercial adhesive DP-460;
- Higher PEI content correlated with a greater reduction in mechanical performance;
- Polar-PCL adhesives evidenced reduced mechanical properties even at low thermoplastic contents, maintaining comparable viscosities to the commercial adhesive;
- Adhesive joints created with Polar-PEI- and Polar-PCL-modified adhesives exhibited superior mechanical performance to those made with commercial adhesive for all the investigated temperatures (i.e., −55 °C, 23 °C, 80 °C);
- The modified epoxy systems enabled the joints’ disassembly and the recycling of adhesives into thermoplastic polymers.

In conclusion, the proposed epoxy-based systems have shown potential for adhesive joint applications, offering the advantages of recyclability and separability. While the addition of PEI ensures good mechanical performance, its use has a considerable environmental impact. Conversely, PCL offers biodegradability, albeit with some mechanical compromises.

Building upon these promising results, future research efforts will focus on the following activities:

- Studying additional adhesive formulations by varying the concentrations of thermoplastic polymers;
- Evaluating the simultaneous addition of thermoplastic polymers to the epoxy resin, thus optimizing PEI/PCL/epoxy resin blends;
- Conducting further investigations, such as peel, double cantilever beam, and end notched flexural tests, to gain a deeper understanding of the adhesive’s mechanical behavior;
- Investigating the mechanical and thermal properties of recycled materials.

## Figures and Tables

**Table 1:** Mechanical properties and chemical composition of aluminum alloy 6061.

Young Modulus [GPa]		Tensile Strength [MPa]		Elongation at Break [%]		Shear Strength [MPa]	
68.9		310		17		207	
Al	Si	Fe	Cu	Mn	Mg	Cr	Zn
95.8–98.6%	0.4–0.8%	0.7%	0.15–0.4%	0.15%	0.8–1.2%	0.04–0.35%	0.25%

**Table 2:** List of the compared epoxy systems.

Code	Hardener [phr]	PEI [phr]	PCL [phr]
Polar	22	0	0
Polar-PCL5	22	0	5
Polar-PCL10	22	0	10
Polar-PCL15	22	0	15
Polar-PCL20	22	0	20
Polar-PEI1	22	1	0
Polar-PEI2	22	2	0
Polar-PEI3	22	3	0
Polar-PEI4	22	4	0
Polar-PEI5	22	5	0

**Table 3:** Main flexural properties of compared polymeric systems.

Code	Flexural Strength [MPa]	Flexural Strength [GPa]	Flexural Strength [%]
DP-460	58.5 ± 3.4	1.5 ± 0.1	7.5 ± 0.7
Polar	96.5 ± 8.1	2.9 ± 0.5	4.8 ± 1.2
Polar-PCL5	40.9 ± 4.5	2.3 ± 0.3	2.0 ± 0.4
Polar-PCL10	34.7 ± 4.7	2.3 ± 0.1	1.7 ± 0.2
Polar-PCL15	35.7 ± 7.3	1.7 ± 0.1	2.9 ± 0.6
Polar-PCL20	42.1 ± 2.2	1.4 ± 0.1	5.0 ± 1.5
Polar-PEI1	95.1 ± 4.6	2.7 ± 0.2	5.2 ± 0.6
Polar-PEI2	98.3 ± 8.3	2.7 ± 0.3	6.8 ± 0.7
Polar-PEI3	83.4 ± 10.2	2.6 ± 0.4	6.0 ± 1.1
Polar-PEI4	76.1 ± 7.4	2.1 ± 0.2	3.6 ± 0.4
Polar-PEI5	47.6 ± 8.2	2.0 ± 0.2	2.5 ± 0.4

**Table 4:** Glass transition temperature (T_g_) values of compared polymeric systems.

Sample	T_g_ [°C]
DP-460	82.3 ± 0.8
Polar	90.9 ± 0.4
Polar-PCL5	78.8 ± 0.4
Polar-PCL10	79.5 ± 0.4
Polar-PCL15	80.2 ± 0.7
Polar-PCL20	78.1 ± 1.0
Polar-PEI1	71.1 ± 1.1
Polar-PEI2	67.5 ± 0.9
Polar-PEI3	70.4 ± 0.7
Polar-PEI4	71.3 ± 1.1
Polar-PEI5	71.4 ± 1.2

## Data Availability

The original contributions presented in this study are included in the article. Further inquiries can be directed to the corresponding author.
